# Unravelling Formaldehyde Metabolism in Bacteria: Road towards Synthetic Methylotrophy

**DOI:** 10.3390/microorganisms10020220

**Published:** 2022-01-20

**Authors:** Vivien Jessica Klein, Marta Irla, Marina Gil López, Trygve Brautaset, Luciana Fernandes Brito

**Affiliations:** Department of Biotechnology and Food Science, Norwegian University of Science and Technology, 7491 Trondheim, Norway; vivien.j.klein@ntnu.no (V.J.K.); marta.k.irla@ntnu.no (M.I.); marina.g.lopez@ntnu.no (M.G.L.); trygve.brautaset@ntnu.no (T.B.)

**Keywords:** formaldehyde, assimilation, dissimilation, methylotrophy, synthetic methylotrophy, regulation systems

## Abstract

Formaldehyde metabolism is prevalent in all organisms, where the accumulation of formaldehyde can be prevented through the activity of dissimilation pathways. Furthermore, formaldehyde assimilatory pathways play a fundamental role in many methylotrophs, which are microorganisms able to build biomass and obtain energy from single- and multicarbon compounds with no carbon–carbon bonds. Here, we describe how formaldehyde is formed in the environment, the mechanisms of its toxicity to the cells, and the cell’s strategies to circumvent it. While their importance is unquestionable for cell survival in formaldehyde rich environments, we present examples of how the modification of native formaldehyde dissimilation pathways in nonmethylotrophic bacteria can be applied to redirect carbon flux toward heterologous, synthetic formaldehyde assimilation pathways introduced into their metabolism. Attempts to engineer methylotrophy into nonmethylotrophic hosts have gained interest in the past decade, with only limited successes leading to the creation of autonomous synthetic methylotrophy. Here, we discuss how native formaldehyde assimilation pathways can additionally be employed as a premise to achieving synthetic methylotrophy. Lastly, we discuss how emerging knowledge on regulation of formaldehyde metabolism can contribute to creating synthetic regulatory circuits applied in metabolic engineering strategies.

## 1. Introduction

Formaldehyde is toxic to all living organisms due to its nonspecific reactions with proteins and nucleic acids, giving rise to the widespread development of mechanisms for its detoxification. However, the focus of this review is formaldehyde metabolism in microorganisms, specifically in bacteria [1]. The primary atmospheric sources of formaldehyde are either anthropogenic (indoor pollution through building materials, vehicle exhaust gases, various combustion sources, and fugitive industrial emissions) or biogenic (live and decaying plants, biomass burning, and seawater) [2,3,4]. Formaldehyde can also be formed as part of bacterial metabolism in biological processes such as the demethylation of lignins or Strecker degradation of glycine with methylglyoxal [5]. High levels of formaldehyde can hamper cell functions due to its cytotoxic effect, which is provoked by its electrophilic reactivity [6]. Formaldehyde forms reactive dihydroxydimethyl peroxides and free radicals in the presence of oxidizing molecules, causing oxidative stress and ultimately cell death [7,8,9,10]. Unspecific binding of formaldehyde to various macromolecules leads to the inactivation of their biological functions [11]. This way, exposure to formaldehyde can damage vital cell components as DNA, RNA, and proteins through processes such as a multistep formation of adducts and cross-links [6,12,13,14,15].

Despite its highly toxic properties, formaldehyde acts as a key intermediate in methylotrophic metabolism directly originating from the oxidation of one-carbon (C1) compounds, excluding rare exceptions [1,6,16,17,18,19,20]. Methylotrophs are microorganisms with the ability to build biomass and obtain energy from either single-carbon compounds such as methane, methanol, or formate, or multicarbon compounds with no carbon –carbon bonds such as dimethyl ether and dimethylamine [20]. All those compounds are commonly known as C1 compounds and will hereafter be referred to as such. Methylotrophs have specifically developed formaldehyde assimilation (i.e., fixation) systems in the course of evolution in order to use formaldehyde for biomass production [1]. Four different cyclic C1 assimilatory pathways have been described for aerobic methylotrophs: formaldehyde fixation can take place via the ribulose monophosphate (RuMP) cycle and the serine cycle in bacteria, while yeasts use the xylulose monophosphate (XuMP) pathway; in a few known methylotrophs, after oxidation of C1 substrates to CO_2_, assimilation of the latter takes place via the ribulose bisphosphate (RuBP) pathway as in classic autotrophic microorganisms [20,21,22]. Because the focus of this review is formaldehyde assimilation in bacteria, the XuMP pathway will not be further explored [23]. Likewise, as anaerobic methylotrophy does not involve formaldehyde and the RuBP pathway relies on autotrophic CO_2_ assimilation, they will also be excluded from this review [23]. Alongside these assimilation pathways, methylotrophs have also developed formaldehyde dissimilation (i.e., oxidation) mechanisms similar to all living organisms in order to cope with excess formaldehyde and preserve their cellular functions [1]. Remarkably, in methylotrophic *Methylorubrum extorquens* (formerly *Methylobacterium extorquens*) AM1, tolerance to formaldehyde is heterogeneously distributed in bacterial cell populations with two distinct gene expression profiles characteristic for tolerant or susceptible cells. While susceptible cells exhibit global stress response to treatment with formaldehyde, the response of the tolerant subpopulation does not seem to involve the formaldehyde oxidation pathway but features a number of chaperones and electron transport chain factors [24]. 

Formaldehyde dissimilation pathways are present not only in methylotrophs but also in nonmethylotrophic microorganisms to prevent the toxic impact of environmental formaldehyde [1]. While for most bacteria, except for methylotrophs, the well-known strategy to maintain intracellular concentrations of formaldehyde at subtoxic levels is its dissimilation, there have recently been attempts at engineering synthetic formaldehyde assimilation pathways into their metabolism using mainly *Escherichia coli* and *Corynebacterium glutamicum* but also *Pseudomonas putida* and most recently *Bacillus subtilis* as host strains [25,26]. Synthetic methylotrophy is based on implementing methylotrophic pathways into nonmethylotrophic platform microorganisms following synthetic biology approaches with only one successful attempt at autonomous methylotrophy so far without the use of cosubstrate, at growth rates of 0.09 h^−1^ [27]. Although employing natural methylotrophs as bioproduction platforms seems to be a more straightforward approach than creating synthetic methylotrophs, it exhibits limitations due to a narrow understanding of their metabolism and physiology and a restricted synthetic biology toolbox [28]. As an alternative strategy, the transfer of methylotrophy into well-established and biotechnologically relevant hosts offers the possibility of a streamlined implementation of C1-based bioproduction processes.

In the past decades, several review papers have described different aspects of formaldehyde metabolism in bacteria and, among others, the following issues were covered: (i) Stress responses of bacterial pathogens to formaldehyde in the environment [19]; (ii) Different cofactor-dependent formaldehyde oxidation pathways in methylotrophic bacteria [29]; (iii) Native methanol metabolism as a foundation for the design of synthetic methylotrophy [30]; (iv) Challenges and opportunities regarding the engineering of unnatural methylotrophic cell factories for methanol- and formate-based biomanufacturing [31,32,33,34,35,36].

**Figure 1:**
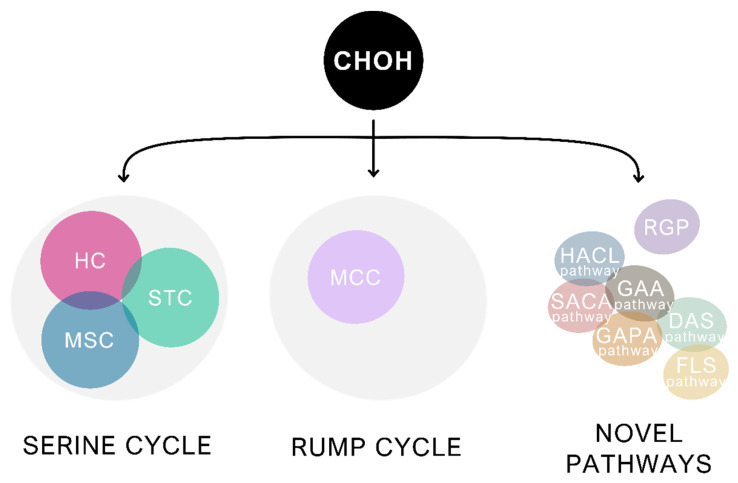
Schematic overview of the native, modified, and novel methylotrophic pathways in bacteria described in this review. CHOH = formaldehyde; HC = homoserine cycle; STC = serine–threonine cycle; MSC = modified serine cycle; MCC = methanol condensation cycle; RGP = reductive glycine pathway; HACL = 2-hydroxyacyl-CoA-lyase; SACA = synthetic acetyl-CoA; GAA = glycolaldehyde assimilation; GAPA = glycolaldehyde-allose 6-phosphate; DAS = dihydroxyacetone synthase; FLS = formolase.

A comprehensive understanding of formaldehyde metabolism and its regulation is indispensable for designing synthetic methylotrophic pathways, which will be further explored in this review. To this end, in addition to the formaldehyde assimilation and dissimilation in native methylotrophs, we will provide examples of formaldehyde dissimilation systems in nonmethylotrophic bacteria given that native methylotrophic routes share multiple enzymes with formaldehyde dissimilatory pathways in nonmethylotrophs. This will be done focusing mainly on chosen organisms used either as gene donors or hosts in attempts at establishing synthetic methylotrophy. For the latter, *E. coli* and *C. glutamicum* will serve as leading examples to showcase the prior attempts to introduce formaldehyde assimilation pathways into nonmethylotrophic bacteria due to their relevance as model organisms and their industrial applications [37,38]. Altogether, we present an exhaustive description of pathways involved in formaldehyde metabolism, both in methylotrophs and nonmethylotrophs, together with examples of how this crucial knowledge has been applied in the establishment of synthetic methylotrophy thus far (Figure 1).

## 2. Understanding and Modification of Formaldehyde Dissimilation Pathways for the Optimization of Synthetic Methylotrophy

Due to the toxic properties of formaldehyde described in the previous Section, its dissimilation is an indispensable feature of bacterial metabolism. Formaldehyde dissimilation in methylotrophic and nonmethylotrophic bacteria relies on either its reaction with sugar phosphates or its oxidation to formate and further to CO_2_, which yields reduction equivalents but does not generate biomass precursors [20]. In this Section, we provide insight into formaldehyde dissimilation pathways and their importance in native methylotrophy (Section 2.1) and show how modification of such dissimilatory pathways in nonmethylotrophic bacteria aids in synthetic methylotrophy efforts (Section 2.2).

### 2.1. Formaldehyde Dissimilatory Pathways in Native Methylotrophs

**Figure 2:**
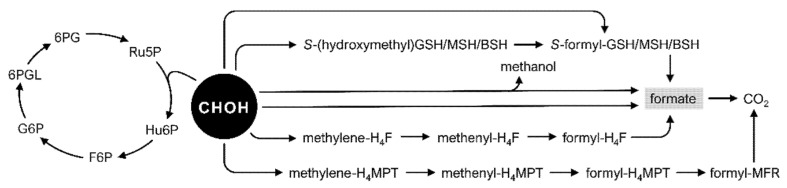
Formaldehyde dissimilation pathways. Schematic view of natural formaldehyde dissimilation pathways mentioned in this review. Metabolites: CHOH = formaldehyde; GSH = glutathione; MSH = mycothiol; BSH = bacillithiol; H_4_F = tetrahydrofolate; H_4_MPT = tetrahydromethanopterin; MFR = methanofuran; Ru5P = ribulose 5-phosphate; Hu6P = hexulose 6-phosphate; F6P = fructose 6-phosphate; G6P = glucose 6-phosphate; 6PGL = 6-phospho-glucono-1,5-lactone; 6PG = 6-phosphogluconate. Relevant metabolites are highlighted in a grey box.

In natural methylotrophs, utilization of C1 compounds starts with their oxidation to the key intermediate formaldehyde prior to either formaldehyde assimilation into central carbon metabolism or its dissimilation [20,21,22]. While formaldehyde dissimilation occurs in both methylotrophic and nonmethylotrophic bacteria by means of cyclic or linear pathways (Table 1 and Table 2, Figure 2), they have two primary functions in methylotrophic bacteria: the control of formaldehyde concentrations below the toxicity threshold and contribution to energy metabolism [20]. The NAD(P)^+^-dependent formaldehyde dehydrogenases (Faldh) require additional cofactors for their activity, such as pterins—tetrahydrofolate (H_4_F) or tetrahydromethanopterin (H_4_MPT), or thiols—glutathione (GSH), mycothiol (MSH), or bacillithiol (BSH) (Figure 2) [22,39,40,41,42,43,44,45,46].

Among the pterin-dependent pathways, the activity of the H_4_F-dependent one (Figure 2) was detected in *B. methanolicus* MGA3 (Table 1) [45,46,47]. After spontaneous condensation of formaldehyde with H_4_F to form methylene-H_4_F, methylene-H_4_F is transformed to methenyl-H_4_F in the reaction catalyzed by methylene-H_4_F dehydrogenase (Figure 2) [45]. The same enzyme catalyzes the conversion of methenyl-H_4_F to formyl-H_4_F, which is followed by two reactions resulting in the formation of formate and its oxidation to CO_2_ [45]. While H_4_F-dependent reactions occur in *M. extorquens* AM1, the H_4_F-dependent enzymes are responsible for maintaining high levels of the intermediates necessary to feed the assimilatory serine cycle with formate representing the primary metabolic branch point between assimilation and dissimilation (Section 3.2) [41,48,49]. The H_4_MPT-dependent dissimilation pathway in *M. extorquens* AM1 begins with the spontaneous condensation of formaldehyde with H_4_MPT to methylene-H_4_MPT, which is accelerated by the formaldehyde-activating enzyme (Fae) (Figure 2) [41,50]. Methylene-H_4_MPT is further converted to methenyl-H_4_MPT in the reaction catalyzed by methylene-H_4_MPT dehydrogenase and followed by several reactions leading to the formation of CO_2_ [41]. A comprehensive analysis was performed to elucidate the distribution of the H_4_MPT-dependent pathway among methylotrophic bacteria, where it was shown that it is present not only in α-proteobacteria that possess the serine cycle for formaldehyde assimilation but also methylotrophic γ- and β-proteobacteria, which assimilate formaldehyde through the RuMP cycle (Table 1, Section 3.1) [39,43,44,51,52]. The H_4_MPT-dependent formaldehyde dissimilation pathway was detected neither in *Amycolatopsis methanolica* nor in *B. methanolicus* MGA3 [39].

In the GSH-dependent pathway, formaldehyde binds to reduced GSH, which leads to the creation of *S*-(hydroxymethyl)GSH, and in *Paracoccus denitrificans,* this spontaneous reaction is accelerated by the glutathione-dependent formaldehyde-activating enzyme (Gfa). However, the presence of Gfa is not essential for the reaction to occur as the GSH-dependent pathway is active in the *gfa*-deficient genetic background [53,54,55,56]. *S*-(hydroxymethyl)GSH is converted to *S*-formyl-GSH by a NAD-GSH-dependent formaldehyde dehydrogenase (GD-Faldh) [40]. *S*-Formyl-GSH is then hydrolyzed by *S*-formyl-GSH hydrolase (Fgh) to GSH and formate, with the latter being subsequently oxidized to CO_2_ (Figure 2) [54]. This pathway is present and functional in different α-proteobacteria, for example, *Methylobacterium aquaticum* 22A, *P. denitrificans, Rhodobacter sphaeroides*, and *Rhodopseudomonas acidophila* (Table 1) [40,51,54,57,58,59,60].

Conversely, the actinobacteria *A. methanolica* and *Rhodococcus erythropolis* use an MSH-dependent formaldehyde dehydrogenase instead of GD-Faldh, whereas *B. methanolicus* MGA3 uses BSH as a cofactor for the oxidation of formaldehyde via *S*-formyl-BSH (Table 1, Figure 2) [45,61,62,63]. In *B. methanolicus* MGA3, this pathway is activated by switching from nonmethylotrophic to methylotrophic growth [45].

In *H. zavarzinii* ZV580 and *Methylococcus capsulatus* Bath, the oxidation of formaldehyde can be catalyzed directly by dye-linked dehydrogenases (DL-Faldh), which most commonly use pyrroloquinoline quinone (PQQ) as their cofactor [64,65]. The PQQ-containing formaldehyde dehydrogenase from *M. capsulatus* Bath is a membrane-bound enzyme coupled to the electron transport chain via a *b*-type cytochrome or a quinone. Furthermore, the PQQ-lanthanide (Ln)-dependent methanol dehydrogenases (Mdh) XoxF1 from *M. extorquens* AM1, *M. aquaticum* 22A, or *Methylacidiphilum fumariolicum* SolV catalyze the oxidation of formaldehyde *in vitro* [51,66,67,68]. However, while in *M. aquaticum* 22A XoxF1 was shown to be functional as a formaldehyde detoxification strategy *in vivo*, the *M. extorquens* AM1-derived XoxF1 does not exhibit that function, and therefore *M. extorquens* AM1 relies on the activity of the H_4_MPT-dependent pathway to maintain formaldehyde levels below the toxicity threshold [51,68]. Strikingly, *M. extorquens* AM1-derived XoxF1 plays a role in regulating its methanol metabolism (Section 4) [69].

As mentioned above, formaldehyde can be dissimilated not only through oxidation in linear dissimilation pathways but also through the reaction with sugar phosphates in a cyclic variant of the RuMP cycle known as cyclic dissimilation or oxidative pentose phosphate pathway (PPP) (Figure 2) [22,44]. This cycle shares the activity of 3-hexulose-6-phosphate synthase (Hps) and 6-phospho-3-hexuloisomerase (Phi) with the formaldehyde assimilation pathway (Section 3.1). In the first step, Hps catalyzes the condensation between formaldehyde and ribulose 5-phosphate (Ru5P) to hexulose 6-phosphate (Hu6P), which is later isomerized into fructose 6-phosphate (F6P) in a reaction catalyzed by Phi [20,22]. In the subsequent reactions, the sugar phosphates undergo a series of chemical transformations to 6-phosphogluconate (6PG) followed by its decarboxylation leading to regeneration of Ru5P (Figure 2) [20]. To our knowledge, this cycle was only found to be active in methylotrophs that utilize the RuMP cycle for formaldehyde assimilation, for example, *B. methanolicus* MGA3, *Methylobacillus flagellatus* KT, and *Methylophaga sulfidovorans* (Table 1) [22,44,70]. The outcome of the cyclic dissimilatory pathway is not exclusively Ru5P regeneration, as it also contributes to formaldehyde detoxification and generation of both energy and reducing power [29]. Interestingly, in *M. flagellatus* KT, this dissimilatory cycle is essential for growth on methanol and probably serves as the primary energy source [44]. Likewise, cyclic dissimilation is one of the main suppliers of NADPH in *B. methanolicus* MGA3 under methylotrophic conditions, highlighting its essential role in cofactor regeneration [71]. Most heterotrophic bacteria rely on the tricarboxylic acid (TCA) cycle and electron transport chain to meet the latter requirement. However, some methylotrophs do not need a complete TCA to fulfil their energy requirements; thus, the cyclic dissimilatory pathway has been proposed as a substitute to the TCA cycle for the generation of reducing power [72]. Taken together, this shows how intertwined C1 metabolic pathways are and that many considerations need to be made while designing pathways to establish synthetic methylotrophy and choosing enzymes with respective activities, which is described in more detail in Section 3.

### 2.2. Modification of Formaldehyde Dissimilation Pathways in Nonmethylotrophic Bacteria Is a Prerequisite for Synthetic Methylotrophy

As mentioned before, formaldehyde dissimilation strategies are ubiquitous in all living organisms. In Section 2.1, we presented examples of such pathways in methylotrophic bacteria where the control of formaldehyde intracellular concentration is particularly important because it is formed as a central metabolite in carbon assimilation processes. Interestingly, formaldehyde dissimilation in nonmethylotrophic bacteria shares multiple enzymes with methylotrophic routes. For instance, the MSH-dependent and the cyclic formaldehyde dissimilation pathway described for methylotrophic bacteria are also present in the nonmethylotrophs *Mycobacterium smegmatis* and *Burkholderia cepacia* TM1 (Table 2) [73,74]. In the latter, the activity of Hps and Phi increases during vanillic acid-based growth compared with other carbon sources, indicating that this pathway is involved in the dissimilation of formaldehyde released during vanillic acid degradation via demethylation [74]. Moreover, many nonmethylotrophs such as *P. putida*, *Pseudomonas aeruginosa*, and *Burkholderia fungorum* LB400 possess GSH-independent formaldehyde dissimilation pathways that involve a zinc-containing Faldh utilizing NAD^+^ as an electron carrier (Table 2) [75,76,77,78]. In fact, *B. fungorum* LB400 has two more formaldehyde oxidation pathways, GSH- and H_4_MPT-dependent (Table 2) [76]. A relatively uncommon formaldehyde detoxification pathway is based on the activity of formaldehyde dismutase present in *P. putida* that catalyzes the dismutation of formaldehyde, leading to the formation of equimolar amounts of the corresponding methanol and formate (Table 2, Figure 2) [79]. *P. putida* acquires its resistance to high concentrations of formaldehyde up to 60 mM through the activity of formaldehyde dismutase, which is induced by supplementation of formaldehyde to the growth medium [79]. Interestingly, putative formaldehyde dismutases in methylotrophic *A. methanolica* and *Mycobacterium gastri* seem not to be involved in formaldehyde detoxification and appear to be active as Mdh, responsible for methanol oxidation [80]. Due to the astounding prevalence and diversity of the formaldehyde dissimilation pathways in nonmethylotrophic bacteria, in this Section, we will focus particularly on three strains: *B. subtilis*, *E. coli*, and *C. glutamicum*, of which *E. coli* and *C. glutamicum* have been used as predominant hosts for synthetic methylotrophy and *B. subtilis* has traditionally been used as a gene donor to create synthetic assimilation pathways.

*B. subtilis,* a cell factory used for microbial production of chemicals, enzymes, and antimicrobial materials, tolerates relatively high doses of formaldehyde with 1–2 mM formaldehyde leading to decreased growth rate but not affecting the number of viable cells [81]. Formaldehyde kills *B. subtilis* spores through DNA damage caused by protein–DNA cross-linking [82]. To circumvent this, the *a*/*b*-type small, acid-soluble spore proteins (SASP) and *β*-subunit of RNA polymerase constitute two active mechanisms in spores as protection against formaldehyde toxicity and mutagenesis [82,83]. In vegetative cells, two formaldehyde dissimilation pathways are active, a thiol-dependent formaldehyde dissimilation pathway and the dissimilatory variant of the RuMP cycle described in Section 2.1 (Table 2) [81,84,85,86]. The expression of *adhA*, encoding a thiol-dependent aldehyde dehydrogenase, is induced by formaldehyde and regulated by AdhR (Section 4) [81]. AdhA has not been functionally characterized yet; however, based on the fact that BSH is the major low molecular mass thiol produced by *B. subtilis*, it is safe to assume that the formaldehyde dehydrogenase in this bacterium is BSH-dependent [84,87]. Formaldehyde induces the expression of two genes, *hxlA* and *hxlB,* that encode Hps and Phi, respectively, essential in formaldehyde dissimilation in *B. subtilis* [85]. Interestingly, the cyclic dissimilation pathway in *B. subtilis* shares the activity of Hps and Phi with both cyclic dissimilation and assimilation pathways present in methylotrophs (Section 2.1 and Section 3.1); however, the fate of F6P is different in these two pathways. Because of this, already more than two decades ago, Yasueda et al. suggested that due to its genetic makeup and presence of Hps-Phi activity, the establishment of synthetic methylotrophy in *B. subtilis* requires theoretically only the additional activity of a heterologous Mdh [85]. Indeed, this approach has been applied very recently as a basis to develop the first methanol-dependent *B. subtilis* [25]. Furthermore, *hxlA* and *hxlB* derived from this bacterium have been routinely applied for heterologous expression in the industrial workhorse for amino acid production *C. glutamicum* in order to drive formaldehyde fixation to F6P in attempts at building synthetic methylotrophy [38,88,89]. In this instance, F6P produced by the action of *hxlA* and *hxlB* enters the process of assimilation catalyzed by enzymes of the nonoxidative PPP, which ultimately leads to methanol-dependent growth and incorporation of methanol-derived carbon (Section 3.1) [88,89].

*C. glutamicum* has two pathways leading to formaldehyde dissimilation, either through direct oxidation to formate by the acetaldehyde dehydrogenase (Ald) or through a series of steps that start with a spontaneous reaction with MSH to form *S*-(hydroxymethyl)MSH (Table 2, Figure 2) [90,91]. For the latter pathway, in the following step *S*-(hydroxymethyl)MSH is oxidized to *S*-formyl-MSH in a reaction catalyzed by AdhE, and then *S*-formyl-MSH is spontaneously hydrolyzed to MSH and formate [90,91,92]. Finally, formate formed by either of the two dissimilation pathways is oxidized to CO_2_ by the formate dehydrogenase FdhF with the contribution of two additional genes, which are presumed to be involved in formate oxidation [90,91,92]. Both Ald and AdhE encoding genes, either single or in combination, have been deleted in *C. glutamicum* in attempts to redirect metabolic flux from formaldehyde dissimilation to assimilation during the establishment of synthetic methylotrophy [88,89,90,93,94,95]. However, deletion of *ald* and *adhE* can lead to adverse effects such as reduced methanol consumption rate, slow growth and a lowered final cell dry weight as compared to the strains harboring *ald* and *adhE*, most probably due to accumulation of formaldehyde above the toxicity threshold [88].

The main formaldehyde dissimilation pathway in *E. coli*, a prominent host for natural product biosynthesis, is GSH-dependent and composed of GD-Faldh (encoded by *frmA*) and Fgh (encoded by *frmB*) (Table 2) [37,96,97,98,99,100]. The genes encoding those two enzymes are clustered and expressed in an operon together with the regulator-encoding gene *frmR* (Section 4). It seems that FrmA is not the unique GD-Faldh in *E. coli,* as it was shown that production of formate also occurs in the Δ*frmA* *E. coli* strain. It was suggested that a promiscuous alcohol dehydrogenase could replace GD-Faldh activity in the conversion of formaldehyde to *S*-formyl-GSH, which is then converted to formate by Fgh (Figure 2) [101]. Moreover, two paralogous genes encode Fgh: *frmB* and *yeiG*. While single deletion of each of those genes did not affect the growth of *E. coli* in the presence of formaldehyde, the double-mutant strain of *E. coli* showed a reduced growth rate in such conditions [99]. In *E. coli* cells, *yeiG* was found to be a constitutively expressed gene, while the expression of *frmB* was a part of the *frmRAB* operon [98,99]. This endogenous system was, in fact, used to achieve synthetic methylotrophy through the oxidation of formaldehyde to formate in *E. coli* combined with formate assimilation by diverse pathways described in more detail in Section 3.2. As mentioned before, the deletion of native formaldehyde dissimilation pathways is a relevant strategy to redirect metabolic flux towards formaldehyde assimilation in synthetic methylotrophs. The deletion of *frmA* or its mutations occurring during adaptive laboratory evolution (ALE) experiments were achieved in several studies to establish synthetic methylotrophy in *E. coli*, which indicates that the conservation of formaldehyde is essential for growth on methylotrophic substrates [27,101,102,103,104,105,106,107,108,109,110,111,112,113,114,115,116,117,118,119,120,121].

## 3. Formaldehyde Assimilation in Methylotrophic Bacteria Is an Inspiration for the Creation of Synthetic Methylotrophs

A detailed understanding of formaldehyde assimilation pathways in methylotrophic bacteria is essential for establishing synthetic methylotrophy. In this Section, we describe in detail the RuMP and the serine cycle as natural formaldehyde assimilation pathways and show several attempts at achieving synthetic methylotrophy using these pathways or their derivatives, as well as the engineering work leading to novel assimilation pathways.

### 3.1. The RuMP Cycle and Its Adaptation to Synthetic Methylotrophy

**Figure 3:**
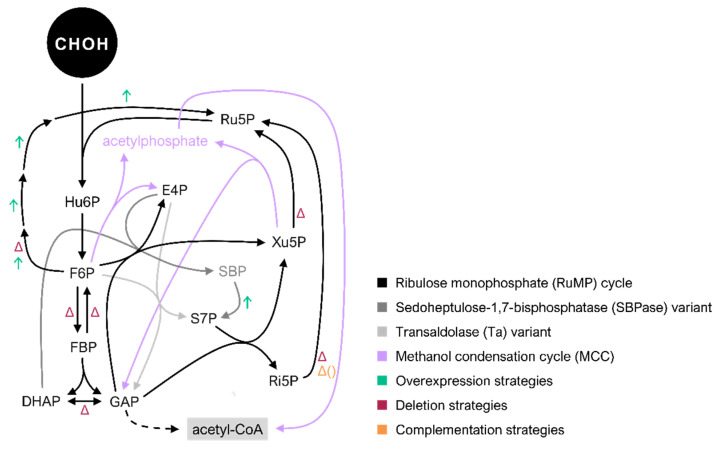
The RuMP cycle and its modifications. Schematic representation of the RuMP cycle (black) with its SBPase variant (dark grey), Ta variant (light grey), and the synthetic biocatalytic MCC (violet). Overexpression strategies are depicted in green, deletion strategies in red, and complementation strategies in orange. Dashed arrows represent multiple reactions. Metabolites: CHOH = formaldehyde; Ru5P = ribulose 5-phosphate; Hu6P = hexulose 6-phosphate; E4P = erythrose 4-phosphate; F6P = fructose 6-phosphate; FBP = fructose 1,6-bisphosphate; DHAP = dihydroxyacetone phosphate; GAP = glyceraldehyde 3-phosphate; S7P = sedoheptulose 7-phosphate; SBP = sedoheptulose 1,7-bisphosphate; Ri5P = ribose 5-phosphate; Xu5P = xylulose 5-phosphate. Relevant metabolites are highlighted in a grey box. Unspecified metabolites leading to Ru5P regeneration through the linear formaldehyde dissimilatory pathway are detailed in Figure 2.

The RuMP cycle is present in bacteria such as *B. methanolicus* MGA3, *M. gastri* MB19, *Nocardia* sp. 239, *A. methanolica*, *M. capsulatus*, *Methylomonas aminofaciens* 77a, and *M. flagellatus* KT [122,123,124,125,126,127,128,129]. Formaldehyde assimilation through the RuMP cycle can be divided into three phases: fixation, cleavage, and rearrangement [20]. As aforementioned, in the fixation phase, Hps catalyzes the condensation between formaldehyde and Ru5P, resulting in the formation of Hu6P (Figure 3) [20]. Hu6P is then isomerized into F6P in a reaction catalyzed by Phi [20]. Subsequently, during the cleavage phase, F6P is phosphorylated to fructose 1,6-bisphosphate (FBP), which is then cleaved to glyceraldehyde 3-phosphate (GAP) and dihydroxyacetone phosphate (DHAP) in the reaction catalyzed by FBP-aldolase [20]. GAP, in turn, can ultimately be converted to acetyl-CoA with pyruvate as an intermediate (Figure 3) [20]. The cleavage phase of the RuMP cycle is followed by the rearrangement phase undergoing the nonoxidative branch of the PPP, with two variants: the fructose 1,6-bisphosphate aldolase/transaldolase (FBPa/Ta) variant and the fructose 1,6-bisphosphate aldolase/sedoheptulose-1,7-bisphosphatase (FBPa/SBPase) variant [20,22,130,131]. In both variants of the pathway, sedoheptulose 7-phosphate (S7P) and GAP molecules are converted to ribose 5-phosphate (Ri5P) and xylulose 5-phosphate (Xu5P) through the activity of transketolase (Tkt) [20]. In the last step of the RuMP cycle, Ru5P is restored either from Ri5P through the activity of Ri5P isomerase (Rpi) or from Xu5P through the activity of Ru5P epimerase (Rpe; Figure 3) [20]. The SBPase variant of the RuMP cycle was shown to be active in the methylotrophic bacterium *B. methanolicus* MGA3 through a study of enzymatic activity and a metabolic flux analysis [71,132]. Nevertheless, according to the fluxomics study conducted by Delépine et al., it cannot be excluded that the Ta variant is operating in parallel. In fact, it was proposed that a parallel activity of both RuMP cycle variants would be beneficial for the growth of *B. methanolicus* MGA3 on a mixture of different carbon sources [71]. Moreover, a recent study detected Ta activity in both *B. methanolicus* MGA3 and PB1 strains and suggested that they may as well use the Ta variant of the RuMP cycle for formaldehyde assimilation [132,133,134].

The RuMP cycle serves as a foundation in the design of synthetic methylotrophy approaches. In the case of synthetic methanol utilization, the common scheme for the implementation of methylotrophy in nonmethylotrophs is the introduction of a minimal set of enzymes: a combination of Mdh that catalyzes the oxidation of methanol to formaldehyde, and Hps and Phi for a two-step irreversible condensation of formaldehyde with Ru5P, following isomerization to form F6P. Mdh that is used to source formaldehyde in the methanol oxidation reaction exhibits formaldehyde reductase activity, which is highly favoured compared to methanol oxidation [135]. For this reason, rapid formaldehyde condensation with Ru5P catalysed by the activity of Hps is necessary to pull the carbon flux towards formaldehyde assimilation. To favour formaldehyde condensation with Ru5P, the effect of spaces between the enzymes and their substrates was considered [103,136,137]. In order to decrease the distance between formaldehyde and Hps, use of the cascade reactions by Mdh, Hps, and Phi was achieved by creating supramolecular enzyme complexes resulting from the fusion of those proteins [103,136]. Accordingly, an *in vitro* fusion protein system consisting of the NAD^+^-dependent Mdh from *B. methanolicus* MGA3 and the Hps-Phi from *M. gastri* enhanced methanol conversion to F6P by promoting efficient formaldehyde channelling through the pathway rather than back to methanol [103]. Likewise, a multienzyme complex composed of the NAD^+^-dependent Mdh from *Geobacillus stearothermophilus* and Hps-Phi from *B. methanolicus* MGA3 showed elevated methanol oxidation activity and F6P formation efficiency [136]. The fate of F6P can be divergent in the bacterial cell; it can either enter the cyclic dissimilatory pathway (Section 2, Figure 2) or be phosphorylated to FBP in a reaction catalyzed by 6-phosphofructokinase, entering the assimilatory pathway (Figure 3). The importance of the deregulation of native formaldehyde dissimilation pathways in strains used as hosts for synthetic methylotrophy to direct the carbon flux towards formaldehyde fixation is discussed in Section 2. 

While it was shown that the minimal catalytic requirement to establish synthetic methylotrophy in microbial species such as *E. coli* and *C. glutamicum* is the introduction of methanol oxidation to formaldehyde by Mdh and its following fixation by Hps and Phi, several additional considerations need to be taken into account. To sustain continuous formaldehyde assimilation, the RuMP cycle must continually regenerate the cosubstrate of Hps, Ru5P. Indeed, the overexpression of genes coding for enzymes that participate in the nonoxidative PPP improved formaldehyde assimilation in *E. coli* [119]. Similarly, seeking to address insufficient Ru5P regeneration in synthetic methylotrophic *E. coli*, Woolston et al. activated the SBPase variant of RuMP in *E. coli* by overexpression of its native *glpX* gene [115]. That, in combination with the supplementation of iodoacetate, an inhibitor of GAP dehydrogenase in lower glycolysis, resulted in increased Ru5P, F6P, and S7P concentrations in *E. coli* cells, which consequently accelerated formaldehyde assimilation [115]. In fact, in a recent work that employed a previously constructed methanol-dependent *C. glutamicum* strain by Tuyishime et al., further ALE experiments in high methanol concentration led to downregulation of glycolysis encoding genes, which improved the regeneration of Ru5P [94,138].

Furthermore, towards the improvement of Ru5P regeneration in the synthetic methylotrophy pathway, the disruption of genes encoding key nonoxidative PPP enzymes such as Rpi or Rpe reroutes the catabolism of cosubstrates xylose or ribose, respectively, to Ru5P formation [89,94,116,139]. The deletion of *pgi* in *E. coli* expressing *hxlAB* derived from *B. subtilis* leads to rerouting glucose carbon flux through the oxidative PPP during methanol co-consumption with glucose [119]. The only successful attempt at autonomous synthetic methylotrophy to date was based on the RuMP cycle [27]. First, the *E. coli* BW25113 background (with a high mutation frequency) was forced towards methanol auxotrophy through deletion of *rpiAB*, provision of xylose as a cosubstrate and heterologous expression of synthetic methylotrophy operon [27]. This strain was evolved for 20 generations, which resulted in the inactivation of genes that would have otherwise led to NADH excess and a loss in formaldehyde levels through its oxidation [27]. Following this, the ensemble modelling for robustness analysis (EMRA) was employed, a tool that suggests enzymes that require up or down-regulation in a given system to avoid the instability caused by kinetic traps, which endorsed the subsequent deletion of *pfkA* encoding phosphofructokinase and replacement of native *gapA* encoding GAP dehydrogenase for the *E. coli* BL21-derived *gapC*, which possesses only 40% of GapA activity [27,140]. That, in addition to *rpiA* complementation to promote utilization of methanol as the sole carbon source and several nutrient weaning strategies, resulted in a successfully generated synthetic methylotroph, albeit with a very low growth rate and biomass yield [27]. It could be observed that a shortage of Ru5P results in formaldehyde accumulation, which in turn leads to formaldehyde-induced DNA-protein cross-linking and, eventually, cell death during the stationary phase [27]. By further ALE of the first synthetic methylotroph, that effect caused by formaldehyde accumulation could be solved by insertion sequence-mediated copy number variations, i.e., as ALE progressed, the copy number of a region spanning 70 kilobases, encompassing the originally introduced synthetic methylotrophy operon, increased [27]. The dynamic adaptation resulted in a final synthetic methylotrophic strain that displayed a doubling time of 8.5 h (growth rate of 0.09 h^−1^) and a maximum optical density at 600 nm of 2 with methanol as sole carbon source [27].

The aforementioned strain also benefited from an ALE-derived 12-basepair deletion in *pgi*, which increased its activity and presumably diverted flux to the oxidative PPP, thus generating additional NADPH for growth [27]. Building on the experience with the optimization through ALE of strains coutilizing methanol and RuMP cycle metabolites, an improved strategy was developed where deletion of RuMP cycle genes is not required to drive co-consumption [141]. In extensive flux balance analysis (FBA), the deletion targets were predicted to obtain genetic backgrounds of *E. coli* that support growth on methanol or methanol together with a multicarbon cosubstrate but not on such cosubstrate alone [141]. Furthermore, genetic makeups lacking the potential for pure methylotrophic growth due to a compromised RuMP cycle were excluded [141]. Considering further selection parameters, two targets for deletion were chosen: *fbp* encoding fructose-1,6-bisphosphatase and *tpiA* encoding triosephosphate isomerase [141]. Deleting each target together with *frm* and plasmid-based expression of *mdh*, *hps*, and *phi* led to the creation of strains that required methanol for growth on pyruvate and are ideal candidates for evolution towards a fully methylotrophic *E. coli* [141].

Balancing metabolic reactions in potential synthetic methylotrophs that do not naturally possess the metabolic landscape present in naturally methylotrophic microorganisms has proven to be challenging. Energy and carbon balance between formaldehyde assimilation and dissimilation in methylotrophs has been gradually optimized through evolution; therefore, artificial introduction of formaldehyde assimilation into nonmethylotrophic species is a desired yet laborious endeavour. For this reason, rather than the plain introduction of formaldehyde assimilation pathways into heterologous hosts, targeted redesign and optimization is required [34]. Several studies that used ALE to achieve synthetic methylotrophy in *E. coli* and *C. glutamicum* revealed causative mutations involved in redox balancing [106,116,138,142]. For instance, a mutation was found in the adenylate cyclase (*cyaA*) gene of *E. coli* [116], CyaA catalyzes the conversion of ATP to cAMP, which subsequently activates the TCA cycle [116]. The deactivation of CyaA results in lower TCA cycle activity, therefore reducing NADH generation by the TCA to balance the NADH generated during exogenous methanol oxidation to formaldehyde by Mdh [116]. In a subsequent *E. coli* work, a mutation was found after ALE in the isocitrate dehydrogenase (*icd*) gene that reduced Icd activity, which ultimately decreased TCA cycle activity balancing intracellular NADH levels [106]. Similarly, an ALE-derived *C. glutamicum* strain showed downregulation of the malate dehydrogenase (Maldh) encoding gene, which led to decreased NADH generation through the TCA cycle, confirming what had been previously observed regarding redox balance in an *E. coli* Δ*maldh* strain [117,138]. In all three cases, the ALE-derived mutations mimic what has been observed in methylotrophic metabolism, namely low TCA cycle activity.

Another way to reduce NADH accumulation is the introduction of production pathways that consume NADH or the conversion of NADH to NADPH for later use in lysine production [102,118,143]. Additionally, lactate dehydrogenase from *E. coli* was employed as an NADH scavenger to favour the Mdh-mediated forward reaction and prevent formaldehyde reduction [103].

The carbon efficiency of the RuMP cycle is not optimal since three formaldehyde molecules are condensed to pyruvate, which is decarboxylated to form acetyl-CoA and CO_2_ [20]. On this account, Bogorad et al. have proposed the construction of the methanol condensation cycle (MCC) (Table 3, Figure 3) [144]. The MCC is a carbon conserved and ATP-independent synthetic biocatalytic pathway that uses enzymatic reactions to convert formaldehyde to acetyl-CoA and water [144]. This pathway modifies the RuMP cycle, which is thereby coupled to a synthetic pathway, the nonoxidative glycolysis (NOG) [145]. The initial steps of the MCC are similar to the RuMP cycle: formaldehyde is combined with Ru5P to form Hu6P, which is further isomerized to F6P (Figure 3) [144]. Then, in the native RuMP cycle, F6P is phosphorylated to FBP and later cleaved to GAP and DHAP. In contrast, in the MCC the NOG takes place instead by employing the activity of a phosphoketolase (Pkt) that can cleave F6P to acetylphosphate and erythrose 4-phosphate (E4P), or Xu5P to acetylphosphate and GAP (which will eventually yield E4P) [144,145,146,147]. The activity of Pkt conserves ATP by phosphorylating the C2 keto group cleaved from F6P or Xu5P using inorganic phosphate [144]. The produced acetylphosphate can be readily converted to acetyl-CoA by a phosphate acetyltransferase (Pta) [144]. Furthermore, the generated E4P reacts with F6P through a series of reactions belonging to the Ta variant of RuMP cycle to regenerate two molecules of Ru5P and complete the MCC (Figure 3) [144]. By avoiding pyruvate decarboxylation to form acetyl-CoA, no carbon is lost in the MCC, which benefits carbon yields in synthetic methylotrophy and, in turn, enhances bioprocess economics. Indeed, the MCC system was successfully used to convert the C1 compound methanol to the higher-chain alcohols ethanol and *n*-butanol. However, the proof-of-concept has been presented in *in vitro* experiments and still awaits application in bacterial cells [144].

### 3.2. The Serine Cycle and Its Derivatives

**Figure 4:**
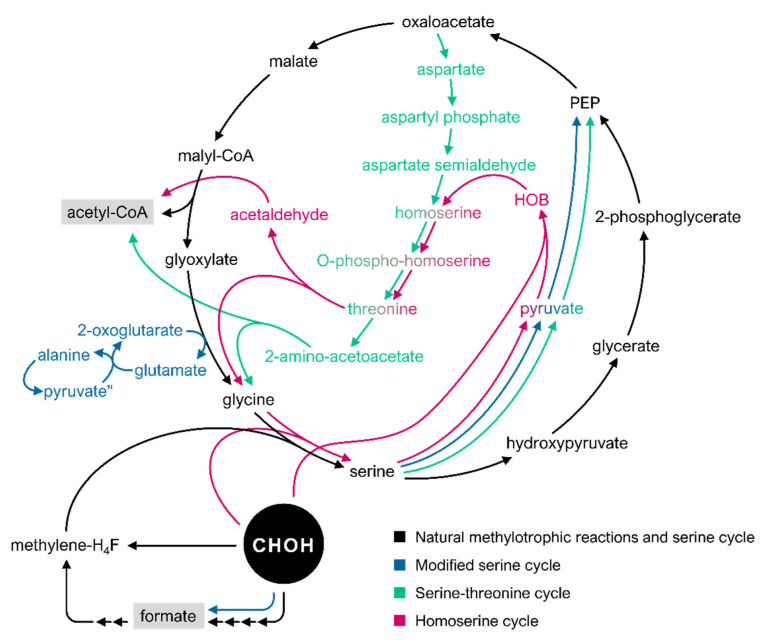
The serine cycle and its modifications. Schematic overview of natural methylotrophic reactions and the serine cycle (black) and its modifications: the modified serine cycle (blue), the serine–threonine cycle (green) and the homoserine cycle (pink). Metabolites: CHOH = formaldehyde; PEP = phosphoenolpyruvate; HOB = 4-hydroxy-2-oxobutanoate; H_4_F = tetrahydrofolate. Some metabolites were duplicated for clearer visualization and are indicated with quotation marks (“). Relevant metabolites are highlighted in a grey box. Unspecified metabolites leading to methylene-H_4_F are part of linear formaldehyde dissimilation and are detailed in Figure 2.

While the RuMP cycle encompasses carbohydrate intermediates, some of which are phosphorylated, the serine cycle requires carboxylic acids and amino acids as intermediates (Table 3) [21]. Formaldehyde enters the serine pathway through methylene-H_4_F that can be produced in two different routes [20,49]. In the first route, formaldehyde and H_4_F are spontaneously combined into methylene-H_4_F, whereas in the second route, formaldehyde is converted to formate in reactions catalyzed by methylene H_4_MPT-dependent enzymes, which is then converted to methylene-H_4_F through the activity of H_4_F-dependent enzymes (Figure 4) [49]. While early research suggested higher metabolic flux through the first of those routes, it was later shown that the latter dominates assimilatory flux in *M. extorquens* AM1 [49,148]. This identifies formate as a metabolic branch point between carbon assimilation and dissimilation pathways in this bacterium (Section 2.1) and highlights the hemiautotrophic nature of the serine cycle where CO_2_ generated through formate oxidation is incorporated into the cycle [49]. The serine pathway begins with the condensation of methylene-H_4_F and glycine to produce serine in the reaction catalyzed by serine hydroxymethyltransferase (Shmt) (Figure 4) [20]. Serine undergoes several reactions catalyzed successively by serine transhydroxymethylase, serine-glyoxylate aminotransferase, hydroxypyruvate reductase (Hpr), glycerate kinase, and enolase to form phosphoenolpyruvate (PEP), which is then carboxylated to malate in a two-step reaction catalyzed by PEP carboxylase and Maldh [20]. Subsequently, malate thiokinase (Mtk) catalyzes the reaction of malate conversion to malyl-CoA, which is thereafter cleaved to form glyoxylate and acetyl-CoA in the reaction catalyzed by malyl-CoA lyase (Mcl) [20]. Glyoxylate is converted to glycine in the following step, and thus the serine cycle is completed (Figure 4) [149].

Theoretically, the serine cycle leads to a 100% carbon yield (the produced glyoxylate eventually regenerates glycine) compared with the RuMP cycle, which has a theoretical carbon yield of 67% due to decarboxylation steps (for each acetyl-CoA synthesized, one molecule of carbon is lost) [34]. Therefore, the serine cycle exhibits a clear asset chemical production of higher carbon compounds such as ethanol, acetone, butyric acid, or terpenoids, with the latter being naturally produced by the serine cycle-utilizing methylotroph *M. extorquens* AM1 [34,150]. Although the MCC pathway successfully circumvents carbon loss in the native RuMP cycle, it is yet to be implemented *in vivo*. The main advantage of the RuMP cycle lies, however, in its energetic efficiency: while NADH and ATP are generated through the RuMP cycle, they are required to drive the process in the serine cycle [34,36]. This is the central reason behind focusing on the RuMP cycle for the majority of synthetic methylotrophy efforts [36]. In addition to this, the RuMP cycle differs from widespread sugar metabolism by only a couple of genes: most nonmethylotrophs possess the required enzymes involved in F6P cleavage and Ru5P rearrangement through the PPP, making the introduction of the missing RuMP cycle modules for formaldehyde fixation much simpler than the several reactions required for transfer of the entire serine cycle [27,34]. Nevertheless, since the achievement of autonomous methylotrophy using the RuMP cycle approach has had limited success, the development of alternative pathways based on the serine cycle has been pursued [36].

One such pathway is the modified serine cycle (Table 3, Figure 4) [151]. In developing this alternative pathway, the goal was two-fold: to reduce the length and complexity of the natural serine cycle and to avoid the deleterious effect of native Hpr in the chosen *E. coli* host [151]. To achieve the first goal, the modified serine cycle uses heterologous Faldh from *P. putida* to catalyze the oxidation of formaldehyde to formate in a single reaction, thus simplifying the four-step oxidation process present in the native pathway (Figure 4) [151]. For the second goal, since side reactivity of Hpr drains intermediate glyoxylate to form glycolate irreversibly at higher catalytic efficiency than it converts hydroxypyruvate to glycerate, the use of the Hpr route was avoided completely [151]. To achieve this, instead of using serine as an amino group donor to convert glyoxylate to glycine, glyoxylate is transaminated with alanine to form glycine in a reaction catalyzed by alanine-glyoxylate transaminase (Agt) from *Saccharomyces cerevisiae* [151]. Glycine is subsequently converted to serine by adding methylene-H_4_F, as in the natural serine cycle. Serine is finally deaminated to pyruvate through serine dehydratase (Sdh) from *Cupriavidus necator,* and PEP is regenerated by the action of endogenous phosphoenolpyruvate synthetase (Pps), thus avoiding the intermediate hydroxypyruvate (Figure 4) [151]. Formaldehyde assimilation via the modified serine cycle was demonstrated through coassimilation of formate or methanol with xylose by isotope labelling experiments [151].

Another variant of the serine cycle is the synthetic serine–threonine cycle (Table 3, Figure 4) [152]. The idea behind the creation of this pathway is to fit the endogenous metabolism of a model host, in this case *E. coli*, in order to avoid introducing non-natural and conflicting fluxes [152]. Moreover, this pathway seeks to avoid interference with central metabolic fluxes, given that the natural serine cycle shares reactions with PPP, glycolysis, and the TCA cycle [110,152]. The *E. coli* genome encodes all the enzymes of the novel pathway except for the formate-tetrahydrofolate ligase (Ftl), which is required to be heterologously expressed in order to assimilate the C1 compound formate [152]. Similar to the modified serine pathway, this adaptation also circumvents the formation of hydroxypyruvate as intermediate; however, serine is here converted to pyruvate by the action of serine deaminase SdaA (Figure 4) [152]. An additional variation is the conversion of oxaloacetate to aspartate catalyzed by aspartate aminotransferase (AspC) for further recycling of glycine via the threonine biosynthesis and cleavage system, which was achieved by overexpression of threonine-cleaving enzymes threonine dehydrogenase (Tdh) and 2-amino-3-ketobutyrate CoA ligase (Kbl). This strategy resulted in high compensatory flux towards threonine biosynthesis, which fueled subsequent glycine production (Figure 4) [152]. By avoiding the conversion of oxaloacetate to malate and further to glyoxylate, the need to heterologously express genes coding for the uncommon enzymes of the natural serine cycle Mtk and Mcl was avoided, succeeding in the goal to adapt the cycle to the endogenous metabolism of *E. coli* [152]. By simultaneous activity of the different pathway sections of the serine–threonine cycle, Yishai et al. successfully achieved formate-dependent growth in *E. coli* strains auxotrophic to the C1-building blocks and serine [152].

Several of the shortcomings displayed by the natural serine cycle, some of them already mentioned, also inspired the design of the homoserine cycle (Table 3, Figure 4) [110]. Three key aspects were tackled in implementing this cycle into the *E. coli* host [110]. Firstly, it was aimed to avoid the competition of flux between the pathway reactions and those of the central metabolism, a shared goal with the serine–threonine cycle [110]. Secondly, the focus was put on reducing the thermodynamic disadvantages of the natural serine cycle [110]. The third aspect was to avoid CO_2_ fixation that follows formaldehyde assimilation since ATP needs to be invested in order to energize carboxylation, and two reduction steps are required to fix CO_2_ in order to bring the carbon to the average oxidation state of carbon in biomass [110]. In order to undertake those issues, He et al. considered characterized promiscuous activities of *E. coli* native enzymes to catalyze all non-natural reactions of the homoserine cycle instead of relying on completely novel reactions [110]. Based on those prerequisites, the homoserine cycle progresses as follows: glycine is directly condensed with formaldehyde to generate serine, catalyzed by the serine aldolase (Sal) reaction, which is promiscuously catalyzed by the *E. coli* threonine aldolase (LtaE) (Figure 4) [110]. This reaction successfully bypasses the long, multicofactor-dependent and ATP-inefficient route for formaldehyde condensation to methylene-H_4_F [110]. After serine is directly converted to pyruvate as described in the serine–threonine cycle, pyruvate is condensed with formaldehyde to generate the non-native metabolite 4-hydroxy-2-oxobutanoate (HOB) by HOB aldolase (Hal) reaction, which is promiscuously catalyzed by *E. coli* 2-keto-3-deoxy-L-rhamnonate aldolase (RhmA) (Figure 4) [110]. HOB is subsequently aminated to homoserine by HOB aminotransferase (Hat), a reaction supported by various endogenous aminotransferases [110]. These reactions effectively replace carboxylation with a formaldehyde assimilation reaction that provides an alternative way to generate a C4 intermediate, with formaldehyde being already at the average oxidation state of cellular carbon [110]. Homoserine is later metabolized by homoserine kinase (Hsk) and threonine synthase (Ts) to produce threonine [110]. Threonine is then cleaved by LtaE that also catalyzes the Sal reaction, to ultimately regenerate glycine and produce acetaldehyde, which will be further oxidized to acetyl-CoA (Figure 4) [110]. The feasibility of the homoserine cycle was demonstrated *in vivo* in several *E. coli* auxotrophic strains whose growth was coupled to the activity of separate homoserine cycle segments [110]. Methanol-dependent conversion of homoserine to glycine and serine and derivation of homoserine and its downstream products from pyruvate and methanol were confirmed individually through ^13^C-labeling experiments [110]. Although these results only confirm the functionality of the homoserine cycle in two separate segments, they are promising first steps towards the establishment of methylotrophic growth via the complete homoserine cycle [110].

### 3.3. Novel Pathways for Assimilation of Formaldehyde

**Figure 5:**
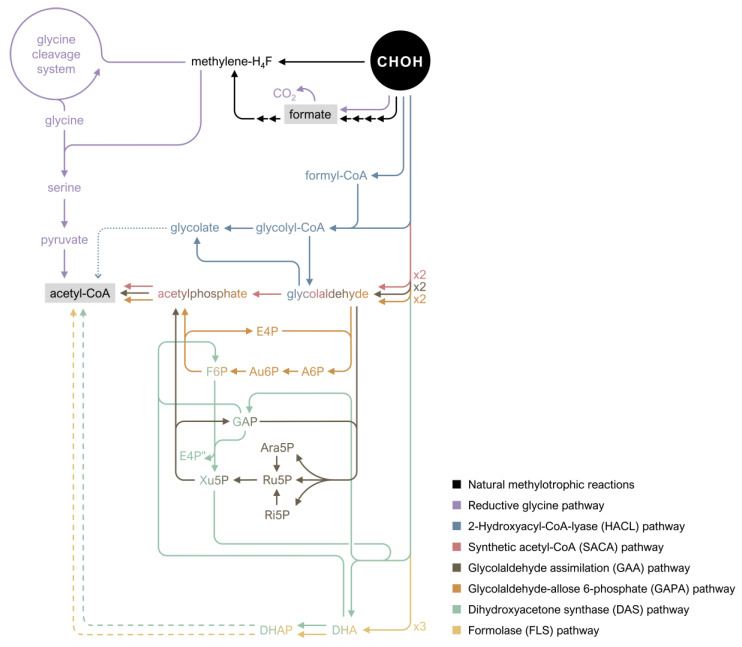
Novel methylotrophic pathways based on natural and synthetic C1-fixing reactions. Schematic overview of the reductive glycine pathway (violet), the HACL pathway (blue), the SACA pathway (red), the GAA pathway (brown), the GAPA pathway (orange), the DAS pathway (green), and the FLS pathway (yellow). Dashed arrows represent multiple reactions. Dotted arrows represent the proposed link to central carbon metabolism. Metabolites: CHOH = formaldehyde; E4P = erythrose 4-phosphate; F6P = fructose 6-phosphate; Au6P = d-allulose 6-phosphate; A6P = 2*R*,3*R*-stereo allose 6-phosphate; GAP = glyceraldehyde 3-phosphate; Xu5P = xylulose 5-phosphate; Ara5P = arabinose 5-phosphate; Ru5P = ribulose 5-phosphate; Ri5P = ribose 5-phosphate; DHA = dihydroxyacetone; DHAP = dihydroxyacetone phosphate; H_4_F = tetrahydrofolate. Relevant metabolites are highlighted in a grey box. Some metabolites were duplicated for clearer visualization and are indicated with quotation marks (“).

In recent times, the development of novel assimilation pathways has received increasing interest, with some of these promising alternative pathways described here. The dihydroxyacetone (DHA) synthase (DAS) pathway is a cyclic formaldehyde assimilation pathway designed using *in silico* modelling [101]. It was built starting with the tool FindPath to identify the most efficient pathway for *E. coli* to consume methanol [101,153]. This resulted in two equally promising hits: a RuMP-based pathway and a hybrid metabolic pathway involving bacterial Mdh from *Acinetobacter gerneri* and yeast DAS from *Pichia angusta,* the latter referred to as the aforementioned DAS pathway (Table 4, Figure 5) [101]. DAS is a transketolase that catalyzes the condensation of formaldehyde with Xu5P yielding GAP and DHA in the XuMP pathway present in methylotrophic yeasts [101]. A library of 266 variants containing different combinations of Mdh and DAS homologues was built and screened using high-throughput ^13^C-labeling experiments [101]. Transcriptional analysis of the expression of genes involved in methanol metabolism indicated that DHA generated from the DAS reaction is subsequently converted to DHAP and Xu5P is regenerated through the activity of F6P aldolase (Fsa), which catalyzes the generation of F6P from GAP and DHA, and TktA which catalyzes the transfer of the C2 keto group from F6P to GAP to form Xu5P and E4P (Figure 5). With the novel pathway, incorporation of 22% methanol carbon was observed in PEP using xylose as cosubstrate, which is similar to values previously reported under comparable cultivation conditions in synthetic methylotrophy attempts in *E. coli* strains expressing the RuMP cycle [101]. Further improvement of the pathway through omics and modelling approaches led to a final optimized strain with a maximum ^13^C-enrichment of 37% in glycerol 3-phosphate [101].

The naturally occurring formaldehyde assimilation pathways and their modifications presented so far are cyclic, require regeneration of formaldehyde acceptors and overlap with central carbon metabolism, making their implementation into nonmethylotrophs challenging in terms of flux balancing between the transferred pathway and its convergence with core metabolism [154]. A different strategy to reshape natural pathways is designing novel, linear pathways based on existing C1-fixing reactions [155]. One example is the reductive glycine pathway, a simple, linear route with small overlap with central metabolism, minimizing requirements in regulatory optimization (Table 4, Figure 5) [156]. The pathway can be divided into four modules: (i) The C1 module, which consists of Ftl, methenyl-H_4_F cyclohydrolase (Fch) and methylene-H_4_F dehydrogenase (MdtA) from *M. extorquens* AM1, converting formate into methylene-H_4_F; (ii) The C2 module, which consists of endogenous *E. coli* host enzymes of the glycine cleavage system (GcvT, GcvH and GcvP), condensing methylene-H_4_F with CO_2_ and ammonia to yield glycine; (iii) The C3 module which consists of native Shmt and serine deaminase, condensing glycine with another methylene-H_4_F to generate serine and ultimately pyruvate; (iv) An energy module, which consists of formate dehydrogenase (Fdh) from *Pseudomonas* sp. strain 101, generating reducing power and energy from formate and thus making this C1 feedstock serve as both carbon and energy source (Figure 5) [156]. After several optimization steps that involve establishing the separate modules for subsequent integration for their combined activity and short-term evolution, Kim et al. successfully generated an *E. coli* strain capable of growing solely on formate and CO_2_ [156]. With the introduction of an additional methanol module by heterologous expression of the *G. stearothermophilus* gene encoding Mdh, growth on a mixture of methanol and CO_2_ could also be achieved, increasing the methylotrophic scope of this pathway [156]. The amino acid labelling patterns detected via ^13^C-labeling experiments confirmed that growth on both conditions indeed takes place via the introduced synthetic route [156].

The development of synthetic alternative formaldehyde assimilation pathways based on non-natural C1-fixing reactions has also received considerable interest [36]. These pathways rely in most cases on enzymes that condense formaldehyde with an additional substrate to generate a relevant metabolite for cell growth [36]. One of the main advantages of these pathways is the lack of need to regenerate the initial formaldehyde acceptors Ru5P or glycine [36]. The so-called formolase (FLS) pathway was the first of such pathways to be developed, where an FLS enzyme was engineered via computational protein design to catalyze the carboligation of three formaldehyde molecules into one DHA molecule, which can enter lower glycolysis via DHAP (Table 4, Figure 5) [157]. In a later study, after several rounds of ALE, ^13^C-labeling experiments showed that the FLS pathway could successfully support methanol-based growth in *E. coli* with the combined activity of Mdh from *B. methanolicus* PB1 and a small amount of yeast extract [113]. This route offers a linear and straightforward way to generate a metabolite that can directly enter central carbon metabolism. However, the catalytic efficiency of FLS is way below that of the average enzyme and still exhibits one-third carbon loss during acetyl-CoA synthesis [36,158].

Another pathway based on formaldehyde condensation is the synthetic acetyl-CoA (SACA) pathway, where a glycolaldehyde synthase (Gals) catalyzes the condensation of two formaldehyde molecules to glycolaldehyde, which is subsequently converted by an *Actinobacteria*-derived Pkt with acetylphosphate synthase activity to acetylphosphate (Table 4, Figure 5). Finally, acetyl-CoA is generated via the action of a Pta, which represents the shortest of the pathways presented in this review [159]. The chosen Gals was designed and engineered to improve its catalytic activity more than 70-fold [159]. The SACA pathway feasibility was first demonstrated *in vitro* by ^13^C-labeled metabolites, which achieved a carbon yield of ~50%, and later verified *in vivo* in *E. coli* using glycolaldehyde, formaldehyde, or methanol as supplementary carbon sources [159]. Even though the SACA pathway is thermodynamically favourable theoretically, surpassing both the MCC and FLS pathway, both Gals and Pkt displayed low substrate affinities *in vivo* [159]. Moreover, acetylphosphate synthase activity by Pkt was inhibited by formaldehyde [159]. This was partially circumvented through the introduction of Mdh to allow for methanol utilization to provide a slow supply of formaldehyde [159]. By this concomitant use of *G. stearothermophilus* Mdh, the addition of ^13^C-methanol resulted in 17% average carbon labelling in PEP, which validated formaldehyde assimilation via the synthetic pathway, albeit contributing only to ~3% biomass from methanol [159].

Similarly, the glycolaldehyde assimilation (GAA) *in vitro* pathway relies on the condensation of two formaldehyde molecules (Table 4, Figure 5). When designing this pathway, Yang et al. predicted 59 ATP-independent and carbon-conserving formaldehyde assimilation pathways using a combinatorial algorithm, the so-called comb–flux balance analysis [160]. The applied algorithm computed multiple optimal pathways in metabolic networks using known reactions from the MetaCyc database and predicted aldolase reactions from the ATLAS database [160]. Interestingly, all 59 pathways contained at least one reaction catalyzed by Pkt, which was previously described when defining the MCC (Section 3.1) and played an important role in the energy-free carbon-conserving conversion of C1 to C2 products [160]. Further evaluation of the pathways led to the selection of those without kinetic traps and the search for enzymes to catalyze the computationally predicted reactions, which ultimately allowed the construction of the GAA pathway [160]. In this *in vitro* multienzyme system, two formaldehyde molecules are first condensed to glycolaldehyde by an engineered Gals as in the SACA pathway (Figure 5) [159,160,161]. Glycolaldehyde is then condensed with GAP by an engineered Ta (TalB^F178Y^) from *E. coli* to form three pentose 5-phosphate compounds: Ri5P, Ru5P and arabinose 5-phosphate (Ara5P), Ara5P being the main product [160,162]. By adding Ara5P isomerase (KdsD), Ara5P can be redirected to Ru5P, and further activity of Rpe converts Ru5P to Xu5P [160]. Finally, Pkt cleaves Xu5P to generate the acetyl-CoA precursor acetylphosphate and regenerate GAP (Figure 5) [160]. After pathway optimization, the synthetic GAA pathway reached 88% product carbon yield [160].

The *in vitro* glycolaldehyde-allose 6-phosphate (GAPA) pathway was developed by the same group that previously constructed the GAA pathway (Table 4, Figure 5) [158]. For this approach, they reused their comb–flux balance analysis but introduced artificially proposed 28 non-natural aldolase reactions not present in the ATLAS database to the known reaction set [158]. This led to the prediction of eight new pathways and two novel aldolase reactions, which ultimately resulted in the construction of the GAPA pathway [158]. This pathway starts with the condensation of two formaldehyde molecules by Gals to yield glycolaldehyde as in both the SACA and GAA pathways (Figure 5) [158]. Glycolaldehyde is subsequently condensed with E4P and converted to 2*R*,3*R*-stereo allose 6-phosphate (A6P) by the novel aldolase reaction catalyzed by DeoC [158]. A6P is then isomerized to d-allulose 6-phosphate (Au6P) by allose 6-phosphate isomerase/ribose 5-phosphate isomerase B (RpiB) and subsequently epimerized to F6P by d-allulose-6-phosphate 3-epimerase (AlsE). F6P is finally hydrolyzed by Pkt similarly to MCC and GAA pathway to produce acetylphosphate and regenerate E4P (Figure 5) [158]. Upon adding all the enzymes to the reaction system, the concentration of acetylphosphate corresponded to a carbon yield of 94% for glycolaldehyde in the GAPA pathway [158].

Another potential strategy for formaldehyde assimilation involves the activity of 2-hydroxyacyl-CoA-lyase (HACL), an enzyme known to be involved in mammalian α-oxidation, that can act reversibly and catalyze the ligation of carbonyl-containing molecules with formyl-CoA to produce C1-elongated 2-hydroxyacyl-CoAs [114]. A prokaryotic variant of HACL from *Rhodospirillales bacterium* with this newfound activity was discovered and functionally expressed in *E. coli* for the first time [114]. The expression of prokaryotic HACL in *E. coli* allowed for ligation of formaldehyde with formyl-CoA to generate the C2 compound glycolyl-CoA via C1 biocatalysis [114]. In order to generate the HACL cosubstrate formyl-CoA, an acyl-CoA reductase from *Listeria monocytogenes* was used for *in situ* formyl-CoA production from formaldehyde [114]. This engineered *E. coli* whole-cell biocatalyst ultimately allowed for the production of glycolate and 2-hydroxyisobutyrate from formaldehyde and formaldehyde and acetone, respectively, achieving up to 84% carbon yield (Table 4, Figure 5) [114,158]. Notably, the HACL pathway allowed for glycolate production using formaldehyde as the sole substrate [114]. Although no cell growth was derived from formaldehyde in the implemented HACL-based biocatalysis, the results presented in this work serve as a valuable proof-of-concept for its further application in synthetic methylotrophy, as the produced glycolate does not only represent a valuable end-product but could also serve as a substrate for subsequent assimilation into central carbon metabolism [114]. Moreover, given that glycolaldehyde is an intermediate, an alternative would be to couple the HACL pathway with the SACA pathway reactions catalyzed by Pkt and Pta, which would yield acetyl-CoA in only two more steps (Figure 5).

While all the aforementioned pathways contribute to promising alternatives theoretically and demonstrate that computational design and enzyme engineering are significant assets for developing competitive synthetic methylotrophy pathways, the poor kinetic properties observed *in vivo* or lack of proof-of-concept in bacterial cells are still a limitation in their applicability today.

## 4. The Understanding of Formaldehyde Metabolism Regulation as a Support to Push Synthetic Methylotrophy

Due to the high toxicity of formaldehyde, its metabolism requires sensitive and fine-tuned regulation systems. Particularly, in methylotrophic microorganisms, formaldehyde metabolism is often regulated through multilevel cascade systems. For example, in the methylotrophic *P. denitrificans*, the formaldehyde metabolism is controlled by a two-component system consisting of FlhR and FlhS (Table 5) [163,164]. The FlhRS system regulates the expression of enzymes that are either involved in production of formaldehyde (Mdh and methylamine dehydrogenase (Madh) or its consumption (GD-Faldh, Fgh) [40,163,164,165]. The FlhRS system is activated, and its expression is induced by the presence of formaldehyde and by the depletion of heterotrophic substrates [163,164]. This regulatory system consists of a signal (FlhS) and a response regulator (FlhR) that binds to DNA [164]. FlhS is a histidine kinase that, when activated through the binding of effector molecules, catalyzes the phosphorylation of FlhR [164]. As soon as FlhR is activated through phosphorylation, it binds to target promoters of C1 metabolism gene clusters, resulting in the expression of genes encoding Fgh and GD-Faldh [163,164]. The upregulation of the expression of these genes takes place during growth on methanol, while they are constitutively expressed at basal levels also during heterotrophic cultivation [164]. Consequently, GD-Faldh and Fgh are synthesized, and thus formaldehyde is converted to CO_2_, which prevents the accumulation of its toxic concentrations [164]. This means that initially, only the formaldehyde-consuming enzymes are synthesized. When formaldehyde concentration increases, the activated FlhR binds to promoters of the operons *mxa* (encoding Mdh) and *mau* (encoding Madh), which, however, does not directly lead to their activation [163,164]. The expression of *mxa* and *mau* is induced by binding the additional regulator MxaX or MauR for expression of *mxa* and *mau*, respectively [164]. The regulatory systems derived from *P. denitrificans* were used to create synthetic regulators that can potentially be applied for dynamic gene expression control in synthetic methylotrophs. For example, the sensoring kinase domains of MxaY or FlhS derived from *P. denitrificans* were fused with the cytoplasmic catalytic domain of the osmosensor histidine kinase EnvZ from *E. coli* in order to create a chimeric sensor histidine kinase that responds to the presence of methanol in the environment [163,166]. A similar approach was used to create a methanol sensor by combining the sensing domain of MxcQ derived from *M. organophilum* XX or *M. extorquens* AM1, or *M. extorquens* AM1-derived MxbD with the transmitter domain of EnvZ from *E. coli* [167,168].

Besides methanol-sensing regulators, *M. extorquens* AM1 possesses a unique, recently discovered formaldehyde sensor enhanced formaldehyde growth protein A (EfgA), which contributes to formaldehyde detoxification not through enzymatic oxidation but relying on binding with this compound [169]. In response to the transient increase of intracellular formaldehyde concentration, EfgA leads to a rapid halt of protein translation and arrest of cell growth which could limit formaldehyde-induced protein damage [169]. Moreover, the changes in the translation are potentially linked to the global transcriptional response to formaldehyde stress mediated by EfgA [170]. Transcriptional response targets biosynthesis of free formaldehyde, contributes to increased formaldehyde consumption by Fae, mitigates proteotoxicity and genotoxicity, and is reversed when formaldehyde concentration decreases [170]. Expression of *efgA* is regulated by TtmR (Table 5), a formaldehyde-responsive MarR family transcription factor, and both EfgA and TtmR are required for the optimal transition from multicarbon to C1 growth [171]. It was shown that heterologous expression of *M. extorquens* AM1-derived *efgA* in *E. coli* increases its formaldehyde resistance, indicating the potential application of EfgA in strategies to increase formaldehyde tolerance during the engineering of synthetic methylotrophy [169].

Methylotrophic growth activates formaldehyde assimilation and dissimilation pathways which should actively control intracellular formaldehyde concentrations. In the facultative methylotroph *B. methanolicus* MGA3 some of the genes involved in the RuMP formaldehyde assimilation cycle are upregulated during growth on methanol versus heterotrophic conditions. However, details of the expression regulation are not elucidated, and the regulator is not yet known. *B. methanolicus* MGA3 cells grown on methanol are more sensitive to formaldehyde than MGA3 cells grown on a nonmethylotrophic substrate [172]. The increased formaldehyde sensitivity during methylotrophic growth might be caused by the already high formaldehyde concentration in the cells grown in methanol and thus the saturation of formaldehyde assimilation and dissimilation pathways in this bacterium. For that reason, supplementation with external formaldehyde can lead to increased formaldehyde toxicity [172]. One of the regulators present in *B. methanolicus* MGA3 is HxlR, which controls the expression of chromosomal genes *hps* and *phi,* upregulated by formaldehyde (Table 5) [133]. It was shown that the introduction of additional copies of *hps* and *phi* through plasmid-based overexpression increases the tolerance of *B. methanolicus* MGA3 cells to high methanol concentrations confirming the central role of that operon in C1 metabolism [133].

The structure of the formaldehyde-responsive transcription factor HxlR was also studied in the nonmethylotroph *B. subtilis* (Table 5). Similarly to TtmR, HxlR belongs to the MarR family of transcription factors, and it controls the expression of *hxlAB* in *B. subtilis* [86,173]. HxlR recognizes formaldehyde through a protein intra-helical cysteine-lysine cross-linking reaction at its N-terminal α1 helix, which in turn leads to a conformational change and transcriptional activation [173]. The resulting intrahelical methylene bridge is a protein modification with a conformational change that allosterically induces transcriptional activation of HxlR [173]. Another regulator responsible for controlling the expression of genes involved in formaldehyde metabolism in *B. subtills* is AdhR (Table 5). AdhR regulates the expression of the BSH-dependent formaldehyde dissimilation pathway in *B. subtilis* and belongs to an NmlR clade within the family of MerR repressor-activators [19]. MerR regulators are sensitive to a wide range of molecules such as soft transition metal ions, the superoxide anion, and drug-like compounds, whereas the members of the NmlR clade respond to oxidative and carbonyl stressors [81,174,175,176]. Similar to other formaldehyde sensors, a cysteine residue is conserved within the NmlR clade; for example, Cys52 is conserved in AdhR from *B. subtilis* [81]. Replacement of this residue with alanine leads to the creation of a strain where *adhA* (*adhC*) is not transcribed in a formaldehyde rich environment [81].

Regulation of expression of the formaldehyde metabolic pathway in *C. glutamicum* is not well characterized. The expression pattern of the gene encoding NAD-linked MSH-dependent formaldehyde dehydrogenase is not known, and neither is its regulation [91]. Expression of *ald* encoding acetaldehyde dehydrogenase that catalyzes the oxidation of formaldehyde depends on the carbon source used for the cultivation of *C. glutamicum*. The activity of Ald increases about 10-fold when ethanol is a carbon source as compared to growth with glucose or mixtures of glucose with ethanol [91,177]. This process is regulated by RamA and RamB and putatively by GlxR (Table 5) [91,177]. Thus, due to differential gene expression, the importance of Ald and FadH might vary depending on the physiological conditions [91]. Methanol catabolism is subject to carbon catabolite repression in the presence of glucose and is dependent on the transcriptional regulator RamA, which was previously shown to be essential for the expression of *adhA* and *ald* [90].

A well-characterized system for the control of formaldehyde metabolism is present in heterotrophic *E. coli*. In this bacterium, *yeiG* encoding Fgh is constitutively expressed, while the expression of Fgh-encoding *frmB,* which belongs to the *frmRAB* operon, increases by 20- to 100-fold over basal levels in the presence of formaldehyde in the environment [98,99]. FrmR is a member of the CsoR/RcnR family of metal ion-sensing transcriptional repressors, which is responsible for controlling the *frmRAB* operon (Table 5) [178,179,180]. In the absence of formaldehyde, FrmR binds to the promoter of the *frmRAB* operon (P*_frm_*), while in its presence FrmR changes its conformation, which leads to the dissociation of the P*_frm_*-FrmR complex [180]. The change of protein conformation is caused by the formation of methylene bridges that link adjacent proline (Pro2) and cysteine (Cys35) residues in the FrmR tetramer [180]. The allosteric mechanism of FrmR is triggered directly by formaldehyde *in vitro* [180]. Sensitivity to formaldehyde requires a cysteine (Cys35 in FrmR) conserved in all DUF156 proteins [181].

As highlighted by the limited success in a full transfer of methylotrophy, and the importance of fine-tuning the C1 metabolic landscape, an important approach to synthetic methylotrophy is the implementation of dynamic formaldehyde regulation mechanisms. The formaldehyde-inducible promoter P*_frm_* was engineered to obtain variants differing in their basal and induced expression levels [182]. A variant of the formaldehyde-responsive promoter characterized with higher basal and induced expression levels compared with P*_frm_* was used for the control of *mdh* and *hxlAB* in a Δ*frmA*Δ*pgi E. coli* genetic background, which led to improved biomass yield in comparison to the strain where the native *E. coli* P*_frm_* was used [182]. Furthermore, using the formaldehyde-inducible promoter P*_frm_* to drive direct regulation of *rpe* and *tkt* genes involved in the regeneration of Ru5P led to significantly improved methanol assimilation into intracellular metabolites in *E. coli* [118]. Global gene regulation is an additional factor that should be considered in establishing synthetic methylotrophy. The use of the non-native substrate methanol for growth likely triggers the response characteristic for nutrient-limiting conditions in *E. coli* [105]. Such response is characterized by diverting resources away from active growth and division in favour of maintenance and stress resistance leading to inhibition of RNA synthesis [105]. This leads to decreased translation and conservation of amino acids concurrent with the upregulation of many amino acid biosynthetic genes [105]. It was shown that the activation of stringent response via overproduction of guanosine tetraphosphate (ppGpp) or enzymes involved in its biosynthesis (RNA polymerase-binding transcription factor DksA and the stress response sigma factor RpoS) enhances methanol utilization in synthetic methylotroph *E. coli* by enabling the biosynthesis of several limiting amino acids using carbon derived from methanol in comparison to the control strain where such amino acids cannot be synthesized [105].

Altogether, a comprehensive understanding of formaldehyde metabolism and its regulation in native methylotrophs is an invaluable asset in designing strategies for its introduction into nonmethylotrophic species. The hitherto research showcases the importance of finding a balance between oxidation of C1 substrates to formaldehyde, endogenous formaldehyde dissimilation, and introduction of synthetic formaldehyde assimilation pathways in order to properly regulate carbon flux towards assimilation and maintain formaldehyde below toxic levels. As exemplified in this review, formaldehyde dissimilation pathways and their regulation seem to be relatively conserved among different bacterial species regardless of their trophic lifestyle, which means that native pathways on nonmethylotrophs can potentially be used in the engineering efforts for synthetic methylotrophy.

## 5. Concluding Remarks and Future Perspectives

Despite the simplicity of the concept, the introduction of synthetic methylotrophy into nonmethylotrophic bacteria has turned out to be a challenging task due to numerous unpredicted constraints. The study and in-depth understanding of native formaldehyde metabolism of methylotrophic strains can offer valuable input to solve those impediments. As discussed in this review, the major factors that affect formaldehyde assimilation are the rate of formaldehyde formation from its C1 precursors, the balance of carbon flux between formaldehyde assimilation and dissimilation, carbon and energy balance yielded by various pathways, and regulation of those processes. Here, the overview of such considerations is provided, serving as a roadmap for future attempts to establish synthetic methylotrophy. A strategy that arises as a seemingly promising alternative is the creation of novel, optimized synthetic pathways designed to circumvent many of the obstacles presented here through the use of optimized enzymes and metabolic shunts.

## Figures and Tables

**Table 1:** List of formaldehyde dissimilation pathways in methylotrophic bacteria.

Pathway ^1^	Characteristic	Example Organism	References
H_4_F-dependent pathway	Linear formaldehyde dissimilation pathway, requires pterin cofactor H_4_F	*B. methanolicus* MGA3	[45,46,47]
H_4_MPT-dependent pathway	Linear formaldehyde dissimilation pathway, requires pterin cofactor H_4_MPT	*M. extorquens* AM1, *Methylobacterium* *organophilum* XX, *M. aquaticum* 22A, *Methylobacterium segetis* 17J42-1^T^*, Hyphomicrobium methylovorum* GM2, *Hyphomicrobium* *zavarzinii* ZV580, *Methylosinus trichosporium* OB3b, *M. capsulatus* Bath, *Methylococcus thermophilus* IIIp, *Methylomicrobium album* BG8, *Methylomonas rubra* 15sh, *M. flagellatus* KT, *Methylophilus methylotrophus* AS	[39,41,42,43,44,51]
GSH-dependent pathway	Linear formaldehyde dissimilation pathway, requires thiol cofactor GSH	*M. aquaticum* 22A, *P. denitrificans, R. sphaeroides, R. acidophila*	[40,51,54,57,58,59,60]
MSH-dependent pathway	Linear formaldehyde dissimilation pathway, requires thiol cofactor MSH	*A. methanolica,* *R. erythropolis*	[61,62,63]
BSH-dependent pathway	Linear formaldehyde dissimilation pathway, requires thiol cofactor BSH	*B. methanolicus* MGA3	[45]
DL-Faldh-mediated formaldehyde dissimilation process	Formaldehyde dissimilation process, relies on activity of DL-Faldh; membrane-associated in *M. capsulatus* Bath	*H. zavarzinii* ZV580, *M. capsulatus* Bath	[64,65]
PQQ-Ln-dependent formaldehyde dissimilation process	Formaldehyde oxidation by a PQQ-Ln-dependent Mdh (XoxF1)	*M. extorquens* AM1, *M. aquaticum* 22A, *M. fumariolicum* SolV	[51,66,67,68]
Dissimilatory variant of RuMP cycle	Cyclic formaldehyde dissimilation pathway	*B. methanolicus* MGA3, *M. flagellatus* KT, *M. sulfidovorans*	[22,44,70]

^1^ Dissimilatory pathways mentioned in this review, their characteristics and example organisms.

**Table 2:** List of formaldehyde dissimilation pathways in nonmethylotrophic bacteria.

Pathway ^1^	Characteristic	Example Organism	Reference
H_4_MPT-dependent pathway	Linear formaldehyde dissimilation pathway, requires pterin cofactor H_4_MPT	*B. fungorum* LB400	[76]
GSH-dependent pathway	Linear formaldehyde dissimilation pathway, requires thiol cofactor GSH	*E. coli*, *B. fungorum* LB400	[76,96,97,98]
BSH-dependent pathway	Linear formaldehyde dissimilation pathway, requires thiol cofactor BSH	*B. subtilis*	[81,84]
MSH-dependent pathway	Linear formaldehyde dissimilation pathway, requires thiol cofactor MSH	*C. glutamicum, M. smegmatis*	[73,90,91]
Faldh dissimilation process	Zinc-dependent formaldehyde oxidation pathway, relies on activity of Faldh that utilizes NAD+ as an electron acceptor	*P. putida*, *P. aeruginosa*, *B. fungorum* LB400	[75,76,77,78]
Formaldehyde dismutase-mediated dissimilation process	Formaldehyde dissimilation based on the activity of formaldehyde dismutase, leading to the formation of equimolar amounts of methanol and formate	*P. putida*	[79]
Ald-mediated dissimilation process	Formaldehyde dissimilation through direct oxidation to formate by Ald	*C. glutamicum*	[38,90,91]
Dissimilatory variant of RuMP cycle	Cyclic formaldehyde dissimilation pathway	*B. subtilis, B. cepacia*	[74,85,86]

^1^ Dissimilatory pathways mentioned in this review, their characteristics and example organisms.

**Table 3:** List of native formaldehyde assimilation pathways in methylotrophic bacteria and their modifications.

Pathway ^1^	Characteristic	Example Organism	Reference
Native pathways			
RuMP cycle	Cyclic formaldehyde assimilation pathway; formaldehyde enters the RuMP cycle through condensation with Ru5P	*B. methanolicus* MGA3*, M. gastri* MB19, *Nocardia* sp. 239, *A. methanolica*, *M. capsulatus*, *M. aminofaciens* 77a, *M. flagellatus* KT	[122,123,124,125,126,127,128,129,132,133,134]
Serine cycle	Cyclic formaldehyde assimilation pathway; formaldehyde enters the pathway through methylene-H_4_F	*M. extorquens* AM1, *M. organophilum* XX, *H. methylovorum* GM2, *M. trichosporium* OB3b	[20,39]
Modified pathways			
MCC	Modified RuMP cycle; synthetic biocatalytic MCC; no carbon loss	Has not been applied *in vivo* yet	[144]
Modified serine cycle	Simplified variant of the serine cycle which uses one step for the oxidation of formaldehyde instead of four in the native serine pathway; avoids the use of the Hpr route by glyoxylate transamination with alanine to form glycine	*E.* *coli*	[151]
Serine–threonine cycle	Synthetic variant of the serine cycle; aims to avoid interference with central metabolic fluxes; circumvents the formation of hydroxypyruvate as intermediate; further recycling of glycine via the threonine biosynthesis and cleavage system	*E. coli*	[152]
Homoserine cycle	Modified variant of the serine cycle; glycine is directly condensed with formaldehyde to generate serine; aims to avoid the competition of flux between the pathway reactions and those of the central metabolism; reduction of thermodynamic disadvantages of the natural serine cycle; CO_2_ fixation is avoided	*E. coli*	[110]

^1^ Assimilatory pathways mentioned in this review, their characteristics and example organisms.

**Table 4:** List of novel methylotrophic pathways.

Pathway ^1^	Characteristic	Host Organism	Reference
Reductive glycine pathway	Linear route that can be divided into four modules; small overlaps with the central metabolism minimizes requirements in regulatory optimization	*E. coli*	[156]
HACL pathway	Synthetic pathway based on the ligation of formaldehyde with formyl-CoA; whole-cell biocatalysis of glycolate	*E. coli*	[114]
SACA pathway	Synthetic linear pathway based on condensation of two formaldehyde molecules using designed Gals	*E. coli*	[159]
FLS pathway	Synthetic pathway in which the computationally designed enzyme FLS catalyzes the carboligation of three formaldehyde molecules	*E. coli*	[113,157]
GAA pathway	Synthetic pathway based on computationally-predicted ATP-independent and carbon-conserving reactions; starts with condensation of two formaldehyde molecules using Gals	Has not been applied *in vivo* yet	[160]
DAS pathway	Synthetic pathway based on bacterial Mdh and yeast DAS identified via *in silico* modelling	*E. coli*	[101]
GAPA pathway	Synthetic pathway based on the introduction of non-natural aldolase reactions; starts with condensation of two formaldehyde molecules using Gals	Has not been applied *in vivo* yet	[158]

^1^ Synthetic pathways mentioned in this review, their characteristics and host organisms.

**Table 5:** List of regulators involved in processes controlling formaldehyde metabolism in bacteria.

Regulator ^1^	Regulated Processes	Example Organism	Reference
FlhRS	Production of formaldehyde (Mdh and Madh) or its consumption (GD-Faldh, Fgh)	*P. denitrificans*	[40,163,164,165]
HxlR	Hps-Phi in RuMP cycle (assimilatory or dissimilatory variant)	*B. methanolicus* MGA3*, B. subtilis*	[133,173]
TtmR	EfgA-mediated formaldehyde stress response	*M. extorquens* AM1	[171]
AdhR	BSH-dependent formaldehyde dissimilation pathway	*B. subtilis*	[19]
FrmR	GSH-dependent formaldehyde dissimilation pathway composed of GD-Faldh and Fgh	*E. coli*	[98,99,178,179,180]
RamAB, GlxR	Ald-mediated formaldehyde dissimilation process	*C. glutamicum*	[91,177]

^1^ Regulators involved in bacterial processes controlling formaldehyde metabolism mentioned in this review, their characteristics and example organisms.

## Data Availability

Not applicable.

## List of Abbreviations

Metabolites	
6PG	6-phosphogluconate
6PGL	6-phospho-glucono-1,5-lactone
A6P	2*R*,3*R*-stereo allose 6-phosphate
Ara5P	arabinose 5-phosphate
Au6P	d-allulose 6-phosphate
BSH	bacillithiol
CHOH	formaldehyde
DHA	dihydroxyacetone
DHAP	dihydroxyacetone phosphate
E4P	erythrose 4-phosphate
F6P	fructose 6-phosphate
FBP	fructose 1,6-bisphosphate
G6P	glucose 6-phosphate
GAP	glyceraldehyde 3-phosphate
GAPA	glycolaldehyde-allose 6-phosphate
GSH	glutathione
H_4_F	tetrahydrofolate
H_4_MPT	tetrahydromethanopterin
HOB	4-hydroxy-2-oxobutanoate
Hu6P	hexulose 6-phosphate
Ln	lanthanide
MSH	mycothiol
PEP	phosphoenolpyruvate
PQQ	pyrroloquinoline quinone
Ri5P	ribose 5-phosphate
RuBP	ribulose bisphosphate
Ru5P	ribulose 5-phosphate
S7P	sedoheptulose 7-phosphate
SBP	sedoheptulose 1,7-bisphosphate
TCA	tricarboxylic acid
Xu5P	xylulose 5-phosphate
XuMP	xylulose monophosphate
Proteins	
Agt	alanine-glyoxylate transaminase
Ald	acetaldehyde dehydrogenase
AlsE	d-allulose-6-phosphate 3-epimerase
AspC	aspartate aminotransferase
CyaA	adenylate cyclase
DAS	dihydroxyacetone synthase
DL-Faldh	dye-linked dehydrogenase
EfgA	enhanced formaldehyde growth protein A
Fae	formaldehyde-activating enzyme
Faldh	NAD(P)^+^-dependent formaldehyde dehydrogenase
FBPa	1,6-bisphosphate aldolase
Fch	methenyl-H_4_F cyclohydrolase
Fdh	formate dehydrogenase
Fgh	*S*-formyl-GSH hydrolase
FlhR	response regulator FlhR
FlhS	signal regulator FlhS
FLS	formolase
Fsa	F6P aldolase
Ftl	formate-tetrahydrofolate ligase
Gals	glycolaldehyde synthase
GD-Faldh	NAD-GSH-dependent formaldehyde dehydrogenase
Gfa	glutathione-dependent formaldehyde-activating enzyme
HACL	2-hydroxyacyl-CoA-lyase
Hal	HOB aldolase
Hat	HOB aminotransferase
HOB	4-hydroxy-2-oxobutanoate
Hpr	hydroxypyruvate reductase
Hps	3-hexulose-6-phosphate synthase
Hsk	homoserine kinase
Icd	isocitrate dehydrogenase
Kbl	2-amino-3-ketobutyrate CoA ligase
KdsD	Ara5P isomerase
LtaE	threonine aldolase
Madh	methylamine dehydrogenase
Maldh	malate dehydrogenase
Mcl	malyl-CoA lyase
Mdh	methanol dehydrogenase
MdtA	methylene-H_4_F dehydrogenase
Mtk	malate thiokinase
Phi	6-phospho-3-hexuloisomerase
Pkt	phosphoketolase
Pps	phosphoenolpyruvate synthetase
Pta	acetyltransferase
RhmA	2-keto-3-deoxy-L-rhamnonate aldolase
Rpe	Ru5P epimerase
Rpi	Ri5P isomerase
RpiB	allose 6-phosphate isomerase/ribose 5-phosphate isomerase B
Sal	serine aldolase
SASP	acid-soluble spore proteins
SBPase	sedoheptulose-1,7-bisphosphatase
Sdh	serine dehydratase
Shmt	hydroxymethyltransferase
Ta	transaldolase
Tdh	threonine dehydrogenase
Tkt	transketolase
Ts	threonine synthase
Pathways	
GAA pathway	glycolaldehyde assimilation pathway
HC	homoserine cycle
MCC	methanol condensation cycle
MSC	modified serine cycle
NOG	nonoxidative glycolysis
PPP	pentose phosphate pathway
RGP	reductive glycine pathway
RuMP cycle	ribulose monophosphate cycle
SACA pathway	synthetic acetyl-CoA pathway
STC	Serine–threonine cycle
TCA cycle	tricarboxylic acid cycle
Other	
ALE	adaptive laboratory evolution
EMRA	ensemble modelling for robustness analysis
FBA	flux balance analysis
